# Recurrent IgG4 Disease-Related Tubulointerstitial Nephritis Treated With Rituximab As Maintenance Therapy

**DOI:** 10.7759/cureus.107911

**Published:** 2026-04-28

**Authors:** Alejandro Valdesuso, Jill Nehrbas, Joseph Dan Khoa Nguyen, Rakesh Malhotra, Tushar Chopra

**Affiliations:** 1 Nephrology, University of Virginia, Charlottesville, USA; 2 Internal Medicine, University of Virginia School of Medicine, Charlottesville, USA; 3 Nephrology, University of Virginia School of Medicine, Charlottesville, USA; 4 Nephrology, University of California San Diego School of Medicine, San Diego, USA

**Keywords:** aki, igg4 disease-related tubulointerstitial nephritis, igg4-related disease, rituximab, tubulo-interstitial nephritis

## Abstract

Immunoglobulin G4-related disease (IgG4-RD) is a rare chronic immune-mediated multisystem disease with renal manifestations of tubulointerstitial nephritis (IgG4-TIN) and membranous nephropathy (IgG4-MN). IgG4-RD and its sequelae are treated with immunosuppressive therapy, including steroids and rituximab (RTX), which have demonstrated high efficacy in achieving disease remission. We describe a patient who was diagnosed with pancreatitis secondary to IgG4-RD through laboratory and imaging studies. Despite being on RTX, he developed acute kidney injury (AKI) and was diagnosed with IgG4-TIN via kidney biopsy. He was treated with prednisone and RTX, and his symptoms and renal function improved. Despite RTX reaching peak efficacy 6 months after administration, our patient had a breakthrough IgG4-TIN during this period. This case highlights the importance of maintaining a high index of suspicion for renal involvement (IgG4-TIN or IgG4-MN) in patients with AKI superimposed on IgG4-RD, and that maintenance therapy with RTX retreatment was associated with longer relapse-free survival at 12 months of follow-up. Our patient had no severe infections or hypogammaglobulinemia (≤5 g/l) with RTX, confirming RTX's safety and efficacy in maintenance remission therapy for recurrent IgG4-TIN.

## Introduction

Immunoglobulin G4-related disease (IgG4-RD) is a rare, multisystemic immune-mediated condition that commonly affects the pancreas, biliary tract, liver, lungs, retroperitoneum, blood vessels, lymph nodes, thyroid, and the kidneys. In the kidneys, the most common manifestation of IgG4-RD is IgG4-related tubulointerstitial nephritis (IgG4-TIN) [1]. IgG4-TIN is characterized by IgG4-positive plasma cell infiltrates within the interstitium, with IgG4-positive plasma cells >10 per high power field in dense areas, and fibrosis with a storiform pattern [1, 2]. IgG4-TIN, similar to IgG4-RD, is most prevalent among men over the age of 50 and has a poorly understood etiology [3]. The disease typically presents with signs of renal insufficiency if the kidneys are the first or only organ to be affected [4]. Treatment of IgG4-TIN is very similar to treatment of IgG4-RD, as it typically responds well to glucocorticoids as first-line therapy [4]. Rituximab (RTX) has also proven to be an effective treatment for both induction therapy and treatment of disease relapse [5, 6]. This case report describes a patient with a history of IgG4-related pancreatitis who developed an acute kidney injury (AKI) due to IgG4-TIN despite already being on RTX therapy. This case highlights the importance of recognition of IgG4-RD as a potential cause of IgG4-TIN. Additionally, it demonstrates the need for awareness of the possibility of IgG4-RD relapse with involvement of the kidneys in those on immunosuppressive therapy.

This article was previously presented as a meeting abstract at the 2024 American Society of Nephrology Annual Meeting on October 25th, 2024.

## Case presentation

A 69-year-old male with a past medical history of eradicated *Helicobacter pylori *infection, B12 deficiency, and hypertension presented to his primary care physician complaining of epigastric pain and postprandial fatigue. He was found to have elevated lipase and was diagnosed with acute pancreatitis. He had no history of excessive alcohol use, gallstones, or severe hyperlipidemia, and no family history of pancreatic or autoimmune disease. He continued to complain of epigastric discomfort, this time accompanied by weight loss. Patient was referred to the gastroenterology clinic, where further workup continued to show elevated liver enzymes, lipase, and IgG4 (Table 1). 

**Table 1:** Table 1. Laboratory trends from initial presentation to gastroenterology clinic to initial presentation to nephrology clinic. AST, aspartate aminotransferase; ALT, alanine aminotransferase; ALP, alkaline phosphatase; IgG4, immunoglobulin G4; Cr, creatinine; UPCR, urine protein-creatinine ratio; UACR, urine albumin-creatinine ratio; C3, complement component 3; C4, complement component 4; KLC, serum free kappa light chain; LLC, serum free lambda light chain; K:L Ratio, kappa lambda-free light chain ratio.

Laboratories (reference range)	Initial Gastroenterology Visit	After Completing Prednisone Taper (3 Months From Initial GI Visit)	Second Flare (6 Months From Initial GI visit)	Third Flare (20 Months From Initial GI Visit)	Initial Nephrology Visit (21 Months From Initial GI visit)	Two Years Into Remission
AST (10-37 U/L)	54 U/L	17 U/L	24 U/L	19 U/L	22 U/L	13 U/L
ALT (5-40 U/L)	96 U/L	16 U/L	21 U/L	17 U/L	27 U/L	14 U/L
ALP (40-125 U/L)	678 U/L	70 U/L	208 U/L	84 U/L	169 U/L	89 U/L
Lipase (7-60 U/L)	163 U/L	40 U/L	10 U/L	-	30 U/L	-
IgG4 (2-96 mg/dl)	882 mg/dl	154 mg/dl	591 mg/dl	158 mg/dl	210 mg/dl	12 mg/dl
Cr (0.8-1.6 mg/dl)	1.1 mg/dl	1.0 mg/dl	1.2 mg/dl	1.5 mg/dl	2.1 mg/dl	1.3 mg/dl
UPCR (<= 0.15 g/g)	-	-	-	-	0.41 g/g	0.07 g/g
UACR (<= 30 mg/g)	-	-	-	-	55.2 mg/g	Not calculable
C3 (82-185 mg/dl)	-	-	-	-	65 mg/dl	125 mg/dl
C4 (15-43 mg/dl)	-	-	-	-	5 mg/dl	35 mg/dl
KLC (0.33-1.94 mg/dl)	-	-	-	-	49.15 mg/dl	-
LLC (0.57-2.63 mg/dl)	-	-	-	-	14.48 mg/dl	-
K:L Ratio (0.26-1.65)	-	-	-	-	3.39	-

He also had diffuse pancreatic thickening with enlarged portacaval and aortocaval lymph nodes on MRI. Pancreatic cancer workup, including CA 19-9, was negative, but he was diagnosed with IgG4-related autoimmune pancreatitis. The patient was treated with a course of prednisone (40 mg daily for 30 days, followed by a 5 mg/week taper over 7 weeks) with significant improvement of symptoms and normalization of liver enzymes as well as lipase. IgG4 level decreased significantly (Table 1).

Three months after completion of the prednisone course, the patient returned to the clinic with worsening fatigue and weakness. Labs showed increased IgG4 and alkaline phosphatase (ALP) (Table 1). However, serum aspartate aminotransferase (AST), ALT, and lipase were within normal range (Table 1). Prednisone was restarted (40 mg daily for 30 days, followed by a 5 mg/week taper), and he was given two infusions of 1 gm RTX, two weeks apart. Patient experienced improvement of GI symptoms; however, he started to experience dyspnea and was found to have saddle pulmonary embolism (PE), for which he was admitted to the intensive care unit. The source of pulmonary embolism was found to be a left deep vein thrombosis (DVT), and the patient underwent catheter-directed thrombolysis and was started on apixaban.

During the following year, the patient received RTX 1 gm every 6 months as part of a maintenance regimen. Just before the second dose of maintenance RTX, he started to experience worsening fatigue, decreased appetite, and epigastric discomfort. No other organ system but the GI system was affected up until this point (third flare) when his creatinine rose from baseline for the first time (Table 1). This new rise in creatinine, as well as frothy urine and new urine odor, prompted nephrology referral.

**Figure 1:**
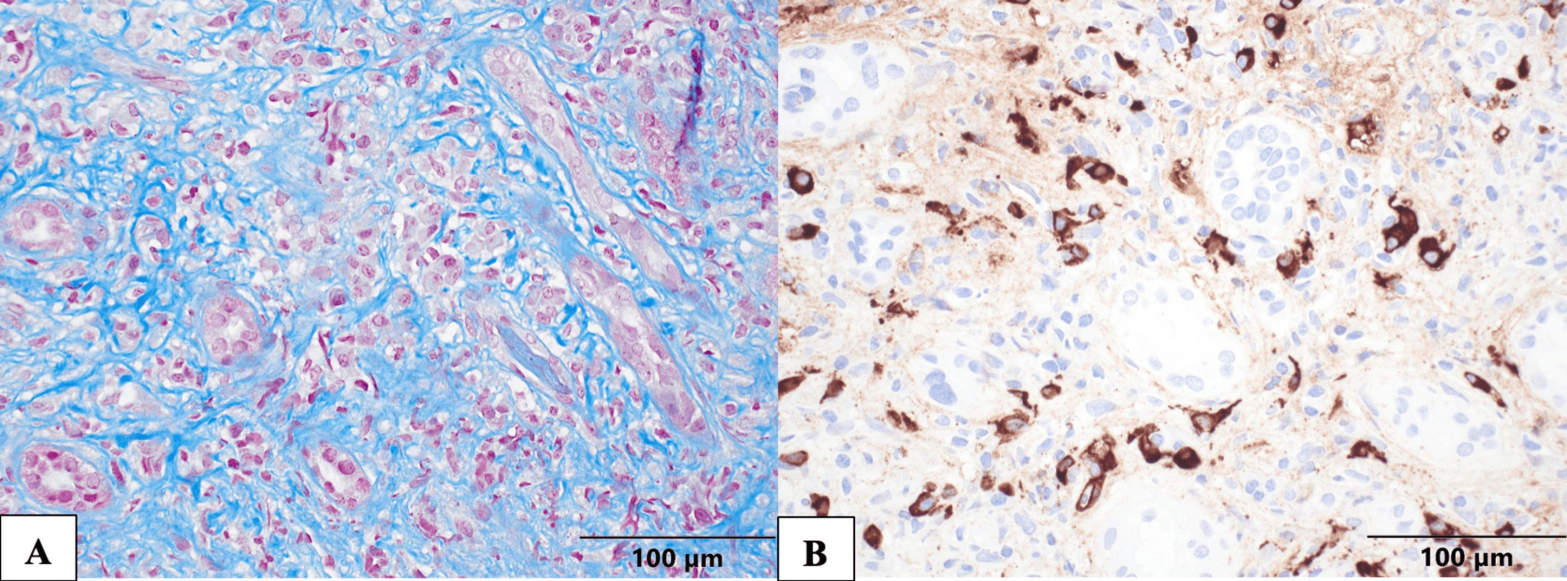
Kidney Biopsy A. The cortex and medulla have severe diffuse fibrosis with "bird’s eye" fibrosis and extensive tubular destruction/dropout and chronic inflammation with numerous lymphocytes and plasma cells and scattered eosinophils. B. Immunohistochemistry (IHC) for IgG4 shows diffusely numerous positive-staining plasma cells with many areas with greater than 10 (often over 50) positive cells per high power field (HPF; 40x objective); focal granular peritubular staining, 2+. IHC for IgG shows comparable numbers of positive cells and focal granular peritubular staining.

Patient was seen at the nephrology clinic, and at the time, he continued to endorse epigastric discomfort, fatigue, and decreased appetite. Physical exam was only significant for mild epigastric tenderness. Urinalysis revealed trace proteinuria and occasional red blood cells (RBCs). Sediment analysis didn’t show any dysmorphic RBCs. Urine protein-creatinine ratio and urine albumin-creatinine ratio were elevated (Table 1). Anti-nuclear antibody (ANA)was mildly elevated at 1:320, but anti-double-stranded DNA (anti-dsDNA)antibodies were not found. C3 and C4 were decreased, and kappa and lambda light chains were elevated, as well as their ratio (Table 1). The rest of the glomerulopathy workup was unremarkable. No peripheral eosinophilia was seen, which was unchanged from prior. MRI performed one week prior to visit had shown kidneys of normal size and location, no renal masses, and no hydronephrosis or hydroureter. To elucidate the cause of the patient’s worsening renal function, a renal biopsy was done, which showed chronic tubulointerstitial nephritis with numerous, mostly IgG4-positive plasma cells, as well as lymphocytes and eosinophils consistent with IgG4-related kidney disease with severe tubulointerstitial fibrosis (Figure 1).

The patient was treated with another course of prednisone (60 mg daily for 7 days, followed by slow taper) and started on a more intense maintenance RTX regimen (two infusions of 1 gm each, two weeks apart, every 6 months), leading to normalization of renal function. He was then switched to annual maintenance RTX (1 gm). For the past 2 years that the patient has been in remission, he has had no proteinuria/albuminuria, C3 and C4 have been normal, and creatinine has remained at 1.2-1.3 mg/dl (Table 1). He follows up at the nephrology clinic every 6 months with a comprehensive metabolic panel, urinalysis (UA), urine protein-creatinine ratio (UPCR), urine albumin-creatinine ratio (UACR), C3, and C4.

## Discussion

Immunoglobulin G4-related disease is a rare and challenging disease to diagnose due to its highly variable presentation, resulting in under-recognition [3]. The kidneys are affected in approximately 15% of cases of IgG4-RD but can be involved in around 30% of cases of IgG4-related pancreatitis [7, 8]. In patients such as ours with IgG4-related pancreatitis, IgG4-related kidney disease is the second most common type of extrapancreatic involvement after cholangitis [9]. While IgG4-TIN is most common, patients can also develop pyelitis, membranous glomerulonephropathy, or hydronephrosis due to retroperitoneal fibrosis [8].

Work-up for IgG4-TIN is often prompted by patients having either imaging abnormalities or unexplained renal dysfunction [10]. On CT imaging, patients can present with diffuse patchy involvement or one or more round or wedge-shaped, low-density lesions in the parenchyma [10]. On serology, patients may present with an elevated serum IgG level (60% cases) and low complement levels, as seen in our case [10]. However, while hypocomplementemia is a characteristic feature, it is absent in 50% of patients diagnosed with IgG4-TIN [10, 11]. IgE and eosinophilia are also often present [10]. For those with evidence of kidney dysfunction and raised serum total protein and total IgG, a renal biopsy is indicated to determine if IgG4-related kidney disease is present [3]. Histopathological findings are typically required for a definitive diagnosis of IgG4-related kidney disease [10]. These findings often include a dense lymphoplasmacytic infiltrate that is rich in IgG4-positive plasma cells and some degree of fibrosis that is organized in a characteristic storiform or swirling pattern [3, 10]. Obliterative phlebitis and tissue eosinophilia may be present as well [3, 10].

All patients with symptomatic IgG4-RD require therapy, and many patients with subclinical disease affecting the kidneys should undergo treatment to prevent irreversible renal damage, according to an international consensus paper on IgG4-RD management [12]. As mentioned in our case, first-line induction therapy for both IgG4-RD and IgG4-TIN is generally a four-week course of glucocorticoids (prednisone 0.6 mg/kg) followed by a steroid taper [13]. In one systematic review that included five observational studies, the mean dose of prednisolone for those with renal involvement was around 40 mg/day [13]. While steroids induce rapid remission in the majority of cases, relapse after treatment is not uncommon [14]. A meta-analysis demonstrated relapse in 64% of IgG4-RD patients who had stopped glucocorticoids and in 36% of those still receiving glucocorticoids [12]. Moreover, approximately 80% of cases treated with steroids are shown to be in remission, with the remaining having a long relapsing and remitting course [15, 16].

In steroid-dependent or steroid-resistant cases, RTX is an alternative treatment option that has demonstrated positive outcomes [6, 17, 18]. RTX has been shown to decrease serum IgG4 and lead to clinical improvement in both IgG4-RD and IgG4-TIN [5, 6, 14]. In a study of 60 IgG4-RD patients, 95% had a clinical response to RTX alone, but 37% experienced relapse following successful treatment [15]. Most patients who relapsed had already experienced B-cell reconstitution with a median time to relapse of 244 days. Elevated baseline serum concentrations of IgG4, IgE, and circulating levels of eosinophils were all associated with a higher risk of relapse following RTX treatment [15]. Interestingly, our patient developed IgG4-TIN after receiving an induction regimen of steroids and RTX, followed by maintenance RTX. 

While a combination of prednisone and a more intense RTX regimen was successful in improving our patient’s renal function, symptoms, and a longer relapse-free survival, this case demonstrates that relapse and even spread of the disease to other organs can still occur despite receiving this combination therapy. Randomized controlled clinical trials are needed to better understand the effectiveness of glucocorticoids and RTX as well as dosing regimens for long-term disease suppression and relapse prevention in both IgG4-RD and IgG4-TIN. Additionally, with recent research implicating CD4+ T cells in the pathogenesis of IgG4-RD, novel treatment approaches that target both B and T lymphocytes could represent a new area of research focus [6, 19].

## Conclusions

This case has three main teaching points: 1) It is important to maintain a high index of suspicion for IgG4-TIN when AKI is superimposed on IgG4-RD and ensure close follow-up for refractory cases; 2) It is possible that IgG4-TIN could develop despite appropriate therapy with steroids and RTX; and 3) A more intense maintenance therapy with RTX (two infusions of 1 gm each, two weeks apart, every 6 months) was associated with longer relapse-free survival at 12 months of follow up in this case. Our patient had no severe infections or hypogammaglobulinemia (≤5 g/l) with RTX, confirming RTX's safety and efficacy in maintenance remission therapy for recurrent IgG4-TIN.

## References

[1] Zhang P, Cornell LD (2017). IgG4-related tubulointerstitial nephritis. Adv Chronic Kidney Dis.

[2] Capecchi R, Giannese D, Moriconi D (2021). Renal involvement in IgG4-related disease: from sunlight to twilight. Front Med (Lausanne).

[3] Momoniat T, Jacob D, Duhli N, Jorna T (2021). IgG4-related tubulointerstitial nephritis. BMJ Case Rep.

[4] Jeong HJ, Shin SJ, Lim BJ (2016). Overview of IgG4-related tubulointerstitial nephritis and its mimickers. J Pathol Transl Med.

[5] Carruthers MN, Topazian MD, Khosroshahi A (2015). Rituximab for IgG4-related disease: a prospective, open-label trial. Ann Rheum Dis.

[6] Ebbo M, Grados A, Samson M (2017). Long-term efficacy and safety of rituximab in IgG4-related disease: data from a French nationwide study of thirty-three patients. PLoS One.

[7] Nada R, Ramachandran R, Kumar A (2016). IgG4-related tubulointerstitial nephritis: a prospective analysis. Int J Rheum Dis.

[8] Wong ET, Lahiri M, Teh M, Leo CC (2019). IgG4-related kidney disease: a curious case of interstitial nephritis with hypocomplementemia. Case Rep Nephrol Dial.

[9] Vujasinovic M, Pozzi Mucelli RM, Valente R, Verbeke CS, Haas SL, Löhr JM (2019). Kidney involvement in patients with type 1 autoimmune pancreatitis. J Clin Med.

[10] Kawano M, Saeki T (2015). IgG4-related kidney disease--an update. Curr Opin Nephrol Hypertens.

[11] Raissian Y, Nasr SH, Larsen CP (2011). Diagnosis of IgG4-related tubulointerstitial nephritis. J Am Soc Nephrol.

[12] Khosroshahi A, Wallace ZS, Crowe JL (2015). International consensus guidance statement on the management and treatment of IgG4-related disease. Arthritis Rheumatol.

[13] Brito-Zerón P, Kostov B, Bosch X, Acar-Denizli N, Ramos-Casals M, Stone JH (2016). Therapeutic approach to IgG4-related disease: a systematic review. Medicine (Baltimore).

[14] McMahon BA, Novick T, Scheel PJ, Bagnasco S, Atta MG (2015). Rituximab for the treatment of IgG4-related tubulointerstitial nephritis: case report and review of the literature. Medicine (Baltimore).

[15] Wallace ZS, Mattoo H, Mahajan VS (2016). Predictors of disease relapse in IgG4-related disease following rituximab. Rheumatology (Oxford).

[16] Saeki T, Kawano M (2014). IgG4-related kidney disease. Kidney Int.

[17] Quattrocchio G, Barreca A, Demarchi A (2018). IgG4-related kidney disease: the effects of a rituximab-based immunosuppressive therapy. Oncotarget.

[18] Khosroshahi A, Bloch DB, Deshpande V, Stone JH (2010). Rituximab therapy leads to rapid decline of serum IgG4 levels and prompt clinical improvement in IgG4-related systemic disease. Arthritis Rheum.

[19] Perugino CA, Stone JH (2020). IgG4-related disease: an update on pathophysiology and implications for clinical care. Nat Rev Rheumatol.

